# Current Trends for Improving Safety of Stereotactic Brain Biopsies: Advanced Optical Methods for Vessel Avoidance and Tumor Detection

**DOI:** 10.3389/fonc.2019.00947

**Published:** 2019-10-02

**Authors:** Serik K. Akshulakov, Talgat T. Kerimbayev, Michael Y. Biryuchkov, Yermek A. Urunbayev, Dara S. Farhadi, Vadim A. Byvaltsev

**Affiliations:** ^1^Department of Neurosurgery, JSC “National Center for Neurosurgery”, Nur-Sultan, Kazakhstan; ^2^Department of Neurosurgery and Traumatology, West Kazakhstan Marat Ospanov State Medical University, Aktobe, Kazakhstan; ^3^University of Arizona College of Medicine, Phoenix, AZ, United States; ^4^Department of Neurosurgery and Innovative Medicine, Irkutsk State Medical University, Irkutsk, Russia

**Keywords:** fluorescence, 5-aminolevulinic acid, stereotactic, spectroscopy, optical, biopsy, fluorescein sodium

## Abstract

Stereotactic brain needle biopsies are indicated for deep-seated or multiple brain lesions and for patients with poor prognosis in whom the risks of resection outweigh the potential outcome benefits. The main goal of such procedures is not to improve the resection extent but to safely acquire viable tissue representative of the lesion for further comprehensive histological, immunohistochemical, and molecular analyses. Herein, we review advanced optical techniques for improvement of safety and efficacy of stereotactic needle biopsy procedures. These technologies are aimed at three main areas of improvement: (1) avoidance of vessel injury, (2) guidance for biopsy acquisition of the viable diagnostic tissue, and (3) methods for rapid intraoperative assessment of stereotactic biopsy specimens. The recent technological developments in stereotactic biopsy probe design include the incorporation of fluorescence imaging, spectroscopy, and label-free imaging techniques. The future advancements of stereotactic biopsy procedures in neuro-oncology include the incorporation of optical probes for real-time vessel detection along and around the biopsy needle trajectory and *in vivo* confirmation of the diagnostic tumor tissue prior to sample acquisition.

## Introduction

Brain needle biopsies are indicated for deep-seated or multiple brain lesions and for patients with poor prognosis in whom the risks of resection outweigh the potential outcome benefits. A recent systematic review and evidence-based clinical practice guideline investigating the role of stereotactic brain biopsy for low-grade gliomas provided level III evidence in support of brain biopsies and recommended that surgeons consider using advanced imaging techniques to improve diagnostic accuracy (1). In suspected low-grade tumors that are not considered for resection, biopsy location should be planned based on molecular guidance techniques, such as positron emission tomography, magnetic resonance (MR) spectroscopy, or others in order to provide a reliable molecular diagnosis (2).

The current standard-of-care method for stereotactic brain needle biopsy involves a 1.6- to 2-mm-diameter needle cannula insertion through a cranial burr-hole aligned to a predetermined trajectory. The two cannulas have overlapping side windows. When the desired position is reached, these windows are aligned, and brain tissue is lodged into cannula using suction and cut by sliding the inner cannula.

Despite the minimally invasive nature of the needle brain biopsy, the limitations and risks are still present and include the following:

1. Non-diagnostic biopsy yield. The frequency of non-diagnostic biopsies ranges in various studies
  ◦ Not-specified non-diagnostic biopsy rate−5.2% (3), 9% (4), 10.7% (5), 13% (6);
  ◦ Biopsy performed without intraoperative frozen section-−11%;
  ◦ Biopsy performed with frozen section on demand−1% (7);
2. Technical failure rate is 2.4–3.7% (7)
3. Complications rate:
  ◦ Overall complications rate−1.2% (3), 7.36% (4);
  ◦ Perioperative−6.3–4.8% (7);
  ◦ Postoperative−10.5–4.8% (7);
  ◦ Hemorrhagic/vessel injury complications: 2.1% (6), 3% (7), 4.35% (4), overall 8.8%, including 1% symptomatic, or >1 cm (8). One study reported the overall rate of hemorrhages on postoperative computerized tomography (CT) as 59.8%, including 41.1% of bleeds <5 mm in diameter and 8.9% of bleeds 3–4 cm in diameter (9).
4. Mortality rate was 0.6% (3), 1.34% (4), and 0.6–3.7% (7) in various studies.

Vessel injury is one of the most feared complications during minimally invasive stereotactic procedures. The rounded tip design of the biopsy needle intends to push aside any vessel encountered along the biopsy needle trajectory. However, such a design does not completely eliminate the potential for vessel injury during the forward movement of the needle. Moreover, the risk of vessel injury is believed to be higher during the side-cutting movement during biopsy acquisition. Therefore, apart from techniques for navigation on the anatomical level, like MR spectroscopy (10, 11), perfusion (12), and metabolism (13), improvements of intraoperative tools for biopsy acquisition and rapid assessment would be beneficial.

This paper aims to provide a concise review of novel fluorescence-based and other optical techniques for improvement of safety and efficacy of minimally invasive stereotactic needle biopsy procedures in neuro-oncology. The main goal of such procedures is not to improve the resection extent but to safely acquire viable tissue representative of the lesion for further comprehensive histological, immunohistochemical, and molecular analyses. Here, we do omit discussion of MR, computed tomography, and ultrasound Doppler (14, 15)-based navigation techniques. Although the potential value of these techniques is undeniable, we focus this review on recently proposed optical methods. The basics of optical techniques are found in recently published reviews of this subject that describe the basics of fluorescence guidance in neuro-oncology (16–21).

## Methods

We performed a literature search in the PubMed database using the terms “biopsy,” “stereotactic,” “fluorescence,” “Raman,” “spectroscopy,” “brain,” “optical,” and “optical probe” in various combinations. Our search included papers published up to November 2018. We did not set a lower bound for this search. The titles were scanned and relevant articles were selected for full-text review, resulting in relevant articles for analysis as presented in the Supplementary Data 1. Additional articles were added from reference lists if deemed relevant. After reviewing the articles, the three key areas for analysis and discussion were selected: (1) avoidance of vessel injury during stereotactic biopsies, (2) probe-based guidance methods for biopsy acquisition, and (3) methods for rapid intraoperative assessment of stereotactic biopsy specimens. The latter two areas were united in one section for discussion due to the similarity in the used optical principles toward the common goal for differentiation of tumor and normal tissues, which can be done *in vivo* (using the miniaturized probes) or *ex vivo* after the biopsy has been acquired (using either miniaturized or benchtop systems).

## Results and Discussion

### Avoidance of Vessel Injury During Stereotactic Biopsy (Table 1)

#### Intravascular Contrast Detection

**Figure 1:**
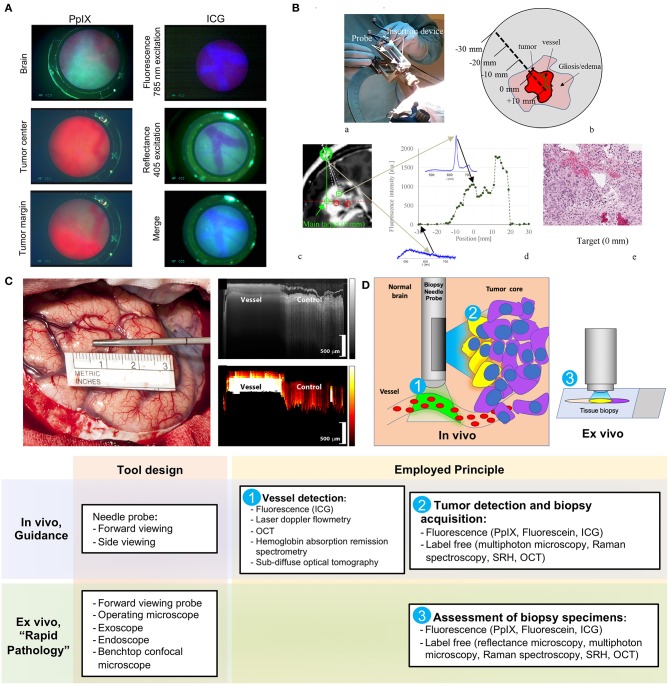
Examples of the optical technologies for brain needle biopsies. **(A)** Images from the fluorescence optical needle endoscope described by Göbel et al. (22) for PpIX visualization in the tumor (left column) and vessel visualization using ICG (right column) in a mouse model. Adapted with permission from Göbel et al. (22)^©^ The Optical Society. **(B)** Illustration of PpIX spectroscopy method for tumor detection during stereotactic biopsy described by Haj-Hosseini et al. (28). The top two panels show the probe positioned in the stereotactic frame and the concept of measurements along the trajectory. The bottom panels show an MR image with calculated targets, spectral data of PpIX along the injection trajectory, and the histopathology slide of the target. Adapted with permission from Haj-Hosseini et al. (28)^©^ The Optical Society. **(C)** Stain-less reflectance imaging method from Ramakonar et al. (27). Left panel shows a photo of an imaging needle rolled over a vessel of 650 μm. The imaging window of the probe is not visible and is facing toward the tissue. The upper right panel displays OCT B-scan consisting of A-scans. The tissue surface corresponds to the top of the image. Depth increases going down the image. The bottom right panel displays a speckle decorrelation image calculated form the OCT scan with high decorrelation as white and low decorrelation as dark red. Adapted from Ramakonar et al. (27) under Creative Commons Attribution license. **(D)** Schematic summary of advanced optical methods and tool designs, for increasing safety of stereotactic brain biopsies. OCT, optical coherence tomography; PpIX, protoporphyrin IX; ICG, indocyanine green; SRH, Stimulated Raman Histology.

Göbel et al. described a small contact forward-viewing endoscopic probe that can fit into a standard biopsy needle and visualize fluorescence signals of protoporphyrin IX (PpIX) and indocyanine green (ICG) (Figure 1A) (22). PpIX is a fluorophore (excitation maximum wavelength is 405 nm; emission maximum, 630 nm) commonly used as the basis of fluorescent-guided neuro-oncology (31), while ICG is a near-infrared fluorophore (excitation maximum wavelength is 780–800 nm; emission maximum, 830 nm) commonly used for vascular flow visualization (32). The multifiber probe includes two excitation diode lasers (405 and 785 nm for PpIX autofluorescence and ICG, respectively) and a charge-coupled device camera for signal detection. This endoscope allowed ICG detection through the brain tissue (about 1 mm thickness) on a phantom model. It also showed reliable detection of a red PpIX fluorescence from the tumor in a mouse glioma model. Subsequently, in a pilot clinical trial (*n* = 1), this needle endoscope was used instead of the standard brain biopsy needle mandarin to visualize fluorescence during probe advancement. The actual biopsy acquisition was performed with a small forward biting biopsy forceps. Although bright PpIX fluorescence from the viable tumor core was visible, no fluorescence in the surrounding needle positions was evident. For vessel visualization, a 200 mg/kg dose of ICG was used in the mouse model. With this, near-infrared ICG fluorescence appeared inside the blood vessels at an excitation of 785 nm (Figure 1A, top panel). With autofluorescence at 405-nm excitation, the vessels appear clearer and darker (Figure 1A, middle panel). With a combination of autofluorescence and ICG visualization simultaneously, the vessels also display clear visualization (Figure 1A, bottom panel). Vessel visualization in a human tissue was not performed.

**Table 1:** Techniques for vessel detection during stereotactic brain biopsies.

**Author, year**	**Technique**	**Clinical data**	**Main findings**	**Limitations**	**Safety**
**FLUORESCENT CONTRAST DETECTION**					
Göbel et al. (22)	1.5-mm-diameter multifiber forward-viewing needle endoscope for dual fluorescence (PpIX, ICG) and autofluorescence imaging. Results in multicolor images.	Pilot clinical trial (*n* = 1) showed feasibility of autofluorescence and PpIX visualization; however, safety, and vessel detection were not evaluated.	Established feasibility on phantom and characterized detection capability of the vessels, normal brain, and viable tumor tissue.	Forward-viewing probe.	Light power was 10 mW.
Rühm et al. (23)	Fiber-based ICG detection in the vessels for stereotactic procedures.	No. Only computer simulation model.	Established safety corridor for excitation light power to prevent normal brain destruction.	Simulation computer model experiment.	Established light intensity safety corridor for ICG.
**STAIN-LESS REFLECTANCE IMAGING APPROACHES**					
Pichette et al. (24)Goyette et al. (25)	24-fiber, 1.7-mm-diameter probe for interstitial sub-diffuse optical tomography. Technology is based on the spectroscopic detection of hemoglobin remittance and sub-diffused light spectra. Creates a 2D map visualizing potential locations of vessels and their proximity to the probe's tip.	No. Only preclinical study on phantom models.	Established feasibility and characterized detection capability of the vessels of various locations and sizes.	Extravascular blood could affect interpretation of the data. Complex probe design.	N/R.
Markwardt et al. (26)	Double fiber-based probe inserted in biopsy needle for hemoglobin remission spectrometry. Allows detection of proximity and size of blood vessels.	No. Only preclinical study on phantom models.	Established feasibility and characterized detection capability of the vessels of various locations and sizes.	Extravascular blood could affect interpretation of the data.	Light power intensity below MPE for the skin of 2 kW/m^2^.
Ramakonar et al. (27)	Side-viewing fiber OCT probe fitting the standard brain biopsy needle with automatic vessel detection on B-imaging mode.	Pilot clinical trial (*n* = 11) demonstrated feasibility of automatic vessel detection.	Established feasibility and characterized detection capability of the vessels.	No forward viewing.	N/R, although considered safe based on the patient data.
**LASER DOPPLER FLOWMETRY**					
Haj-Hosseini et al. (28)	9-fiber, forward-viewing probe fitting the biopsy needle for simultaneous laser Doppler flowmetry and PpIX fluorescence spectral detection.	Similar probes for laser Doppler flowmetry imaging have been studied in stereotactic procedures under IRB approval in previous studies (29, 30).	Established feasibility and characterized brain perfusion along the biopsy needle trajectory.	No side viewing.	Light power 10 mW for PpIX excitation.

*ICG, indocyanine green; N/R, not reported; PpIX, protoporphyrin IX; ICG, indocyanine green; OCT, optical coherence tomography; MPE, maximal permissible exposure*.

Ruhm et al. investigated forward-viewing microfiber probe for ICG detection using a computer simulation of ICG fluorescence excited and detected through the same fiber-optic system in homogeneous human brain tissue (23). Although detection of intravascular ICG is widely used in open vascular neurosurgery (33), its application for detection of deep vessels might not be optimal due to the fast redistribution and ICG fluorescence decay. Leakage of blood or trace amounts of blood containing ICG in the vicinity of the probe would result in false-positive measurements. Furthermore, the dosage and timing of ICG administration would need to be optimized, and the necessity for additional drug injection should be considered, especially when comparing to other methods for vessel detection.

### Laser Doppler Flowmetry

Another recently reported tool is a 2.2-mm-diameter forward-viewing needle probe that combines fluorescence spectral detection and laser Doppler flowmetry (28). The probe was designed to fit the Leksell® Stereotactic System. By measuring the frequency shift in the 780-nm backscattered laser light caused by the cell movements in the capillaries, the device assesses brain perfusion and blood flow. The pilot study on three patients demonstrated reliable detection of PpIX spectra (405 excitation laser) along the trajectory of the device (Figure 1B). Although increased perfusion was detected in two biopsy locations, no information regarding the changes in the surgical plan or needle trajectory was reported (28). A similar stand-alone forward-viewing laser Doppler flowmetry needle probe had been investigated by the same group during the electrode placement for deep brain stimulation in patients (29, 30). Although the main goal of these studies was to establish the link between the measured blood flow and anatomy along trajectories, the authors did encounter one bleeding episode evidenced by significantly increased blood flow measures, which was later confirmed by CT (29). Subsequently, the authors suggested that this method could detect small vessels and thus decrease the risk of bleeding complications in stereotactic procedures (30). Obtaining measurements every 0.5 mm along the injection trajectory was proposed; however, it would result in a significant increase of surgical time (20 s per measurement resulting in at least 33.3 min for 5-cm trajectory) (28). Additionally, this probe has not been integrated into a brain biopsy needle for side view assessment. Future studies are necessary to assess if vascular imaging is helpful to guide intraoperative adjustments of the surgical plan and prevent vessel injury and bleeding.

### Stain-Less Reflectance Imaging Approaches

Ramakonar et al. reported the performance of a side-viewing probe that fit a standard side-cutting brain biopsy needle for optical coherence tomography (OCT) imaging and differentiation of solid tissue and vessels (Figure 1C) (27). This imaging method is based on the detection and reconstruction of backscattered light from the 1,300-nm near-infrared illumination light, allowing 1–1.5 mm penetration depth and 5–20 μm resolution. OCT imaging was presented in an earlier paper by Kut et al. that demonstrated the modality's efficacy as a label-free technique for differentiating cancer from non-cancer in human brain tissues at a 1- to 1.5-mm penetration depth (34). To overcome the challenge of OCT's shallow depth, Ramakonar et al. was able to develop an optically guided biopsy needle capable of OCT imaging in order to visualize blood vessels at greater tissue depths. The information is then displayed on a monitor as a B-scan acquired during needle movements. Initial analysis of surface cortical vessels in patients that underwent craniotomy showed that the device detected blood vessels with a diameter of >500 μm with a sensitivity of 91.2% and a specificity of 97.7% (27). Furthermore, the authors validated these findings by demonstrating deep brain vessel detection capability (vessels were preselected on MRI) during brain needle biopsies in three patients (27).

Markwardt et al. in a phantom model and in *ex vivo* porcine brains demonstrated that a side-viewing double fiber probe for remission spectrometry inserted in a side-cutting biopsy needle can determine the proximity of the vessel to the needle (26). The method is based on the illumination of the tissue with a light from a broad wavelength light emitting diode (LED) light source (400–700 nm) and spectroscopic analysis of the remitted light. The remitted light is assessed at wavelengths that are characteristic of hemoglobin absorption (578 and 650 nm for oxygenated and deoxygenated, respectively), allowing the estimation of the proximity of the vessels to the probe. The method results are displayed as ratio values that represent the proximity, size, and orientation of the hemoglobin-containing vessels to the probe. Although there is no visual information regarding the appearance of the tissue, the technique is relatively simple and inexpensive.

Pichette et al. in phantom experiments demonstrated that interstitial sub-diffuse optical tomography technology can detect vessels with diameters of more than 300 μm for up to 2 mm from the biopsy needle (24). The 1.7-mm-diameter probe consists of 24 side-viewing fibers that provide circumferential scanning and detection of the spectrally resolved remitted light from the tissue around the needle guide. The main advantage of this method is that the probe scans the whole volume of tissues surrounding the needle, creating a two-dimensional visual signal map across the depth and circumference position. An additional advantage is higher tissue penetration depth when compared to optical coherence tomography (2 vs. ~1 mm) (24). Remission spectrometry and sub-diffuse optical tomography both rely on the hemoglobin absorption spectra and do not require additional contrast agents (24, 26).

## Methods for Biopsy Acquisition Guidance and for Rapid Intraoperative Assessment of Obtained Specimens

### Identification of Viable Diagnostic Tissue

Decreasing the number of stereotactic biopsies represents another strategy to minimize the risk of brain and vascular damage. Therefore, technologies that allow for the detection of viable tumor tissue along the biopsy needle trajectory are valuable. Such techniques allow not only to increase accuracy for diagnostic sample acquisition for proper histopathological diagnostics but also to avoid unnecessary repetitive needle biopsies.

### Methods Based on the Detection of Molecular Labels in the Tumor Tissue

The presence of 5-aminolevulinic (5-ALA)-induced PpIX fluorescence is diagnostic for malignant tumors and can be used to assess stereotactic biopsy samples before submitting them to pathology, while the absence of fluorescence can filter out necrotic areas and inflammatory reactive tissue without malignant cells. This method was confirmed in multiple studies (35–40). Detection of PpIX fluorescence was highly diagnostic for viable tumor tissue in patients with intracranial lymphomas (41), high-grade gliomas, and anaplastic foci in patients with low-grade gliomas (42). Fluorescence positive samples might even not require intraoperative frozen section analysis and could be sent directly for a permanent section because of the high positive predictive value of PpIX fluorescence (39, 43, 44). In cases of low or negative fluorescence, the intraoperative frozen section analysis is recommended with subsequent biopsies upon the results of the intraoperative histopathological analysis (43). Excitation of PpIX at 633 nm can further increase the diagnostic utility of such a method, as it allows for deeper imaging through a small layer of blood or tissue, which was previously impossible with 405-nm excitation (45, 46).

Similarly to 5-ALA, fluorescein sodium can be used to assess stereotactic biopsy specimens under the special fluorescence mode of the surgical microscope. High-grade glioma tissue shows strong yellow fluorescence when excited at about 488 nm (47). Being a relatively inexpensive drug, fluorescein sodium could also be visualized using a low-cost miniature device, Fluoropen, which is essentially a LED torch with a blue filter mounted at the light source for excitation and a yellow filter attached around the torch as a cone collar (48). The device is positioned close to the specimen and yellow fluorescence is observed through the yellow filter (48). Observation of fluorescein fluorescence from the specimens had a 100% positive predictive value and a 25% negative predictive value of a lesional tissue (49). Overall, the fluorescein-based method was shown to be as effective as frozen section analysis for the diagnostic biopsy screening (49).

ICG may also be used as a highlighter of the tumor tissue (50–54). Although there are few reports about the ICG use for tumor detection during stereotactic biopsies (16), open surgical visualization showed high sensitivity but low specificity of the second-window ICG (52). Other targeted molecular labels that can be used for open fluorescence-guided surgery, for example, BLZ-100, an ICG-conjugated tumor-targeting peptide chlorotoxin used for imaging, also hold potential for stereotactic needle biopsy procedures (55).

Apart from the macroscopic identification of the retained fluorescent drug, the obtained stereotactic biopsy specimens could be subjected to analysis with other methods alternative to a frozen section. Such methods might be less laborious and time-consuming. Stereotactic samples could be stained with rapid fluorophores *ex vivo* and scanned with a confocal microscope (56, 57). Miniaturized handheld confocal laser endomicroscopy with fluorescein sodium injected intraoperatively as a contrast can visualize the histological architecture of brain biopsies *ex vivo* and *in vivo* (58–62). However, this probe has not been assessed in stereotactic procedures.

A thin forward-viewing fiber-based confocal laser endomicroscope that can fit biopsy needles is available for 488-nm and 660-nm excitable fluorophores (fluorescein sodium and ICG, respectively) (63, 64). A pilot study has demonstrated that such an endomicroscope with fluorescein as a contrast can visualize brain tumor architecture during a stereotactic brain biopsy procedure in humans (65). A similar technique was tested by Lynagh et al. in a proof-of-concept study for the detection of fluorescein and blue-fluorescence protein labeled glioma cells in a rat model using a 0.65-mm fiber microendoscope coupled with a clinical stereotactic biopsy needle (66). Microendoscopy technology is similar to a needle-based contact endoscope (22) and other fiber probes for spectral measurements that were discussed above (28, 35, 44), but is able to provide fluorescence tissue image with higher resolution down to the cellular level.

### Label-Free Methods

Various microscopy imaging methods that are not dependent on the drug-induced contrast and instead rely on intrinsic optical properties of the tissues recently received clinical attention. Such methods could be used for rapid intraoperative assessment of the acquired stereotactic brain biopsies to increase diagnostic yield and improve pathology workflow.

It has been shown that measurements of reflectance and fluorescence spectra of unstained brain tissues can differentiate radiation necrosis in brain tumor tissue (67). Reflectance confocal microscopy can differentiate viable brain tumor tissue from necrotic tissue and further characterize histological appearance with high diagnostic accuracy (68, 69). Label-free multiphoton microscopy methods were successfully used for the imaging of Alzheimer's disease brain samples (70). Raman microspectroscopic microscopy imaging has been studied for histological assessment of brain tumor biopsies (71). A notably successful variant of Raman microscopy is the Stimulated Raman Histology (SRH) technique, which has been used for *ex vivo* histological assessment of brain tumor biopsies. SRH provided high-resolution digital images that look similar to the standard hematoxylin and eosin staining (72, 73). SRH is a stand-alone imaging system that can be positioned in the operating room for rapid intraoperative pathological assessment (72, 73). Although most of the imaging studies were performed on benchtop microscopes, some multiphoton microscopy modes (CARS and TREF) will be available in endoscopes and needle-size probes in the near future (74).

Stevens et al. described a 1.8-mm-diameter forward-viewing 830-nm Raman-based spectroscopy probe without fibers that fit a standard brain biopsy needle (75, 76). The new design of the probe resulted in significant noise reduction compared to the silica-fiber-based Raman imaging and demonstrated discrimination of various porcine brain structures including white matter, gray matter, and blood vessels based on the Raman spectra (75).

## Summary

Current developments for increasing the safety and efficacy of stereotactic brain biopsy procedures are centered around the two main areas: avoidance of the vessels and detection of the viable diagnostic tissue, which could be achieved *in vivo* or *ex vivo* (Figure 1D).

Because vessel avoidance is a major component for the safety of any stereotactic needle-based procedure including needle brain biopsy, deep brain stimulation (77), or interstitial laser thermal therapy (78, 79), advancements in the surgical tools that allow for timely vessel detection is of utmost importance. Several technologies for vessel detection implemented into a biopsy needle included detection of fluorescent intravascular contrast, laser Doppler flowmetry, optical coherence and interstitial sub-diffuse tomography, and remission spectrometry. Non-fluorescence methods based on the blood flow and hemoglobin detection are thought to be the most promising as they avoid dealing with fluctuations in the intravascular contrast concentrations after injection. It should be noted that the current literature does not provide definitive evidence on the efficacy of such methods for vessel injury avoidance; therefore, more clinical studies are necessary.

*Ex vivo* confirmation of PpIX or fluorescein fluorescence from biopsy samples has been established to have a high positive predictive value of a diagnostic biopsy in multiple studies and can be recommended as a routine method for stereotactic biopsy at this current stage. However, the possibility for identification of the correct biopsy needle position *in vivo* prior to actual biopsy acquisition with a small optical tool is even more exciting. Most of such reports are based on the detection of the intra-tumoral fluorescent molecules (PpIX, fluorescein, and ICG); however, the development of reflectance and multiphoton microscopy techniques and militarization of microscopes would allow label-free tumor tissue identification through a biopsy needle in the future. There is a marked diversity in qualitative and quantitative optical methods for brain tumor tissue identification, and each of them requires a significant learning curve and dedication. So far, the most progress has been achieved with 5-ALA-induced PpIX, which is highly specific for malignant tissues and can be detected spectroscopically with a single optical fiber inserted in the biopsy needle.

Another limitation is that most of the discussed methods require timely interpretation and adjustments of multiple parameters during the procedure to ensure optimal performance, which is not always practical and may lead to information overload. For example, results of quantitative spectroscopy are usually displayed on the screen in a graph form. This information could be converted into an auditory format, which would be easy to understand and non-disruptive during the surgery (80). Finally, the possibility of light-induced tissue damage should be carefully considered when developing novel optical and fluorescent tools for *in vivo* diagnostics.

When considering the design of an ideal probe, it should incorporate vessel detection as well as tumor detection modules, most likely in forward and side views simultaneously for the safest possible probe insertion and biopsy acquisition. Machine learning, coupled with optical visualization technologies, would be a basis for computer-aided, real-time tissue image analysis for the selection of the best probe trajectory or location for biopsy acquisition. Besides these technologies that are used for vessel avoidance and tumor detection, future development of probes could also include augmented or virtual reality for clinicians to perform more accurate probe trajectory planning.

## Author Contributions

All authors listed have made a substantial, direct and intellectual contribution to the work, and approved it for publication.

### Conflict of Interest

The authors declare that the research was conducted in the absence of any commercial or financial relationships that could be construed as a potential conflict of interest.

## Abbreviations

PpIX: protoporphyrin IX
ICG: indocyanine green.
