# Supplementary material for: Current Trends for Improving Safety of Stereotactic Brain Biopsies: Advanced Optical Methods for Vessel Avoidance and Tumor Detection
Source: Front Oncol. 2019 Oct 2;9:947. doi: 10.3389/fonc.2019.00947 (PMC6783564; doi:10.3389/fonc.2019.00947)
Supplement: Supplementary file 1 [file Data_Sheet_1.pdf]

Supplemental data 1.

Literature search

Pubmed(22/01/19): Key words: “biopsy, stereotactic, fluorescence” – 53 results – 16 results

Pubmed(22/01/19): Key words: “biopsy, stereotactic, RAMAN” – 14 results – 2 selected

Pubmed(22/01/19): Key words: “biopsy, stereotactic, spectroscopy, brain” – 142 results – 14 selected

Pubmed(22/01/19): Key words: “biopsy, stereotactic, optical” – 51 results – 9 selected

Pubmed(22/01/19): Key words: “Biopsy, optical probe, brain” – 110 results – 29 selected

List of papers:

1: Desroches J, Lemoine É, Pinto M, Marple E, Urmey K, Diaz R, Guiot MC, Wilson BC, Petrecca K, Leblond F. Development and first in-human use of a Raman spectroscopy guidance system integrated with a brain biopsy needle. J Biophotonics. 2019 Jan 12. doi: 10.1002/jbio.201800396. [Epub ahead of print] PubMed PMID: 30636032.

2: Ramakonar H, Quirk BC, Kirk RW, Li J, Jacques A, Lind CRP, McLaughlin RA. Intraoperative detection of blood vessels with an imaging needle during neurosurgery in humans. Sci Adv. 2018 Dec 19;4(12):eaav4992. doi: 10.1126/sciadv.aav4992. eCollection 2018 Dec. PubMed PMID: 30585293; PubMed Central PMCID: PMC6300404.

3: Wu L, Yu H, Zhou R, Luo J, Zhao J, Li Y, Wang K, Wang Y, Li H. Probe-based confocal laser endomicroscopy for diagnosis of nasopharyngeal carcinoma in vivo. Laryngoscope. 2018 Aug 27. doi: 10.1002/lary.27450. [Epub ahead of print] PubMed PMID: 30151887.

4: Li D, Zhang J, Chi C, Xiao X, Wang J, Lang L, Ali I, Niu G, Zhang L, Tian J, Ji N, Zhu Z, Chen X. First-in-human study of PET and optical dual-modality image-guided surgery in glioblastoma using (68)Ga-IRDye800CW-BBN. Theranostics. 2018 Apr 3;8(9):2508-2520. doi: 10.7150/thno.25599. eCollection 2018. PubMed PMID: 29721096; PubMed Central PMCID: PMC5928906.

5: Markwardt NA, Stepp H, Franz G, Sroka R, Goetz M, Zelenkov P, Rühm A. Remission spectrometry for blood vessel detection during stereotactic biopsy of brain tumors. J Biophotonics. 2017 Aug;10(8):1080-1094. doi: 10.1002/jbio.201600193. Epub 2016 Oct 7. PubMed PMID: 27714967.

6: Anand S, Cicchi R, Giordano F, Conti V, Buccoliero AM, Guerrini R, Pavone FS. Multimodal fiber-probe spectroscopy allows detecting epileptogenic focal cortical dysplasia in children. J Biophotonics. 2017 Jun;10(6-7):896-904. doi: 10.1002/jbio.201600136. Epub 2017 Jan 9. PubMed PMID: 28067998.

7: Markwardt NA, Haj-Hosseini N, Hollnburger B, Stepp H, Zelenkov P, Rühm A. 405 nm versus 633 nm for protoporphyrin IX excitation in fluorescence-guided stereotactic biopsy of brain tumors. J Biophotonics. 2016 Sep;9(9):901-12. doi: 10.1002/jbio.201500195. Epub 2015 Nov 13. PubMed PMID: 26564058.

8: Stevens OA, Hutchings J, Gray W, Vincent RL, Day JC. Miniature standoff Raman

probe for neurosurgical applications. J Biomed Opt. 2016 Aug 1;21(8):87002. doi: 10.1117/1.JBO.21.8.087002. PubMed PMID: 27533445.

9: Blanco VM, Chu Z, LaSance K, Gray BD, Pak KY, Rider T, Greis KD, Qi X. Optical and nuclear imaging of glioblastoma with phosphatidylserine-targeted nanovesicles. Oncotarget. 2016 May 31;7(22):32866-75. doi: 10.18632/oncotarget.8763. PubMed PMID: 27096954; PubMed Central PMCID: PMC5078058.

10: Benoit a la Guillaume E, Martins F, Boccara C, Harms F. High-resolution handheld rigid endomicroscope based on full-field optical coherence tomography. J Biomed Opt. 2016 Feb;21(2):26005. doi: 10.1117/1.JBO.21.2.026005. PubMed PMID: 26857471.

11: Dochow S, Ma D, Latka I, Bocklitz T, Hartl B, Bec J, Fatakdawala H, Marple E, Urmey K, Wachsmann-Hogiu S, Schmitt M, Marcu L, Popp J. Combined fiber probe for fluorescence lifetime and Raman spectroscopy. Anal Bioanal Chem. 2015 Nov;407(27):8291-301. doi: 10.1007/s00216-015-8800-5. Epub 2015 Jun 21. PubMed PMID: 26093843; PubMed Central PMCID: PMC4995092.

12: Haj-Hosseini N, Richter JC, Hallbeck M, Wårdell K. Low dose 5-aminolevulinic acid: Implications in spectroscopic measurements during brain tumor surgery. Photodiagnosis Photodyn Ther. 2015 Jun;12(2):209-14. doi:

10.1016/j.pdpdt.2015.03.004. Epub 2015 Mar 26. PubMed PMID: 25818546.

13: Lee JH, Kim DH, Song WK, Oh MK, Ko DK. Label-free imaging and quantitative chemical analysis of Alzheimer's disease brain samples with multimodal multiphoton nonlinear optical microspectroscopy. J Biomed Opt. 2015 May;20(5):56013. doi: 10.1117/1.JBO.20.5.056013. PubMed PMID: 26021718.

14: Romeike BF, Meyer T, Reichart R, Kalff R, Petersen I, Dietzek B, Popp J. Coherent anti-Stokes Raman scattering and two photon excited fluorescence for neurosurgery. Clin Neurol Neurosurg. 2015 Apr;131:42-6. doi: 10.1016/j.clineuro.2015.01.022. Epub 2015 Jan 31. PubMed PMID: 25688033.

15: Tedford CE, DeLapp S, Jacques S, Anders J. Quantitative analysis of transcranial and intraparenchymal light penetration in human cadaver brain tissue. Lasers Surg Med. 2015 Apr;47(4):312-22. doi: 10.1002/lsm.22343. Epub 2015 Mar 13. Erratum in: Lasers Surg Med. 2015 Jul;47(5):466. PubMed PMID: 25772014.

16: Xie H, Xie Z, Mousavi M, Bendsoe N, Brydegaard M, Axelsson J, Andersson-Engels S. Design and validation of a fiber optic point probe instrument for therapy guidance and monitoring. J Biomed Opt. 2014 Jul;19(7):71408. doi: 10.1117/1.JBO.19.7.071408. PubMed PMID: 24623193.

17: Yang C, Hou VW, Girard EJ, Nelson LY, Seibel EJ. Target-to-background enhancement in multispectral endoscopy with background autofluorescence mitigation for quantitative molecular imaging. J Biomed Opt. 2014;19(7):76014. doi: 10.1117/1.JBO.19.7.076014. PubMed PMID: 25027002; PubMed Central PMCID: PMC4098034.

18: Kim JK, Choi JW, Yun SH. Optical fine-needle imaging biopsy of the brain. Biomed Opt Express. 2013 Nov 15;4(12):2846-54. doi: 10.1364/BOE.4.002846. eCollection 2013. PubMed PMID: 24409385; PubMed Central PMCID: PMC3862157.

19: Cappon DJ, Farrell TJ, Fang Q, Hayward JE. Fiber-optic probe design and optical property recovery algorithm for optical biopsy of brain tissue. J Biomed Opt. 2013 Oct;18(10):107004. doi: 10.1117/1.JBO.18.10.107004. PubMed PMID: 24121732.

20: Krafft C, Belay B, Bergner N, Romeike BF, Reichart R, Kalff R, Popp J. Advances in optical biopsy--correlation of malignancy and cell density of primary brain tumors using Raman microspectroscopic imaging. Analyst. 2012 Dec 7;137(23):5533-7. doi: 10.1039/c2an36083g. Epub 2012 Oct 10. PubMed PMID: 23050263.

21: Göbel W, Brucker D, Kienast Y, Johansson A, Kniebühler G, Rühm A, Eigenbrod S, Fischer S, Goetz M, Kreth FW, Ehrhardt A, Stepp H, Irion KM, Herms J. Optical

needle endoscope for safe and precise stereotactically guided biopsy sampling in neurosurgery. Opt Express. 2012 Nov 19;20(24):26117-26. doi: 10.1364/OE.20.026117. PubMed PMID: 23187467.

22: Agnes RS, Broome AM, Wang J, Verma A, Lavik K, Basilion JP. An optical probe for noninvasive molecular imaging of orthotopic brain tumors overexpressing epidermal growth factor receptor. Mol Cancer Ther. 2012 Oct;11(10):2202-11. doi: 10.1158/1535-7163.MCT-12-0211. Epub 2012 Jul 17. PubMed PMID: 22807580; PubMed Central PMCID: PMC3829608.

23: Sanai N, Eschbacher J, Hattendorf G, Coons SW, Preul MC, Smith KA, Nakaji P, Spetzler RF. Intraoperative confocal microscopy for brain tumors: a feasibility analysis in humans. Neurosurgery. 2011 Jun;68(2 Suppl Operative):282-90; discussion 290. doi: 10.1227/NEU.0b013e318212464e. PubMed PMID: 21336204.

24: Sankar T, Delaney PM, Ryan RW, Eschbacher J, Abdelwahab M, Nakaji P, Coons SW, Scheck AC, Smith KA, Spetzler RF, Preul MC. Miniaturized handheld confocal microscopy for neurosurgery: results in an experimental glioblastoma model. Neurosurgery. 2010 Feb;66(2):410-7; discussion 417-8. doi: 10.1227/01.NEU.0000365772.66324.6F. PubMed PMID: 20087141.

25: Giller CA, Liu H, German DC, Kashyap D, Dewey RB. A stereotactic near-infrared probe for localization during functional neurosurgical procedures:

further experience. J Neurosurg. 2009 Feb;110(2):263-73. doi: 10.3171/2008.8.JNS08728. PubMed PMID: 19012484.

26: Bhatia S, Ragheb J, Johnson M, Oh S, Sandberg DI, Lin WC. The role of optical spectroscopy in epilepsy surgery in children. Neurosurg Focus. 2008 Sep;25(3):E24. doi: 10.3171/FOC/2008/25/9/E24. PubMed PMID: 18759626.

27: Lin WC, Mahadevan-Jansen A, Johnson MD, Weil RJ, Toms SA. In vivo optical spectroscopy detects radiation damage in brain tissue. Neurosurgery. 2005 Sep;57(3):518-25; discussion 518-25. PubMed PMID: 16145531.

28: Boppart SA, Brezinski ME, Pitris C, Fujimoto JG. Optical coherence tomography for neurosurgical imaging of human intracortical melanoma. Neurosurgery. 1998 Oct;43(4):834-41. PubMed PMID: 9766311.

29: Germano IM, Queenan JV. Clinical experience with intracranial brain needle biopsy using frameless surgical navigation. Comput Aided Surg. 1998;3(1):33-9. PubMed PMID: 9699077.

1: Markwardt NA, Stepp H, Franz G, Sroka R, Goetz M, Zelenkov P, Rühm A. Remission spectrometry for blood vessel detection during stereotactic biopsy of brain tumors. J Biophotonics. 2017 Aug;10(8):1080-1094. doi:

10.1002/jbio.201600193. Epub 2016 Oct 7. PubMed PMID: 27714967.

2: Markwardt NA, Haj-Hosseini N, Hollnburger B, Stepp H, Zelenkov P, Rühm A. 405 nm versus 633 nm for protoporphyrin IX excitation in fluorescence-guided stereotactic biopsy of brain tumors. J Biophotonics. 2016 Sep;9(9):901-12. doi: 10.1002/jbio.201500195. Epub 2015 Nov 13. PubMed PMID: 26564058.

3: Stevens OA, Hutchings J, Gray W, Vincent RL, Day JC. Miniature standoff Raman probe for neurosurgical applications. J Biomed Opt. 2016 Aug 1;21(8):87002. doi: 10.1117/1.JBO.21.8.087002. PubMed PMID: 27533445.

4: Göbel W, Brucker D, Kienast Y, Johansson A, Kniebühler G, Rühm A, Eigenbrod S, Fischer S, Goetz M, Kreth FW, Ehrhardt A, Stepp H, Irion KM, Herms J. Optical needle endoscope for safe and precise stereotactically guided biopsy sampling in neurosurgery. Opt Express. 2012 Nov 19;20(24):26117-26. doi: 10.1364/OE.20.026117. PubMed PMID: 23187467.

5: Sankar T, Delaney PM, Ryan RW, Eschbacher J, Abdelwahab M, Nakaji P, Coons SW, Scheck AC, Smith KA, Spetzler RF, Preul MC. Miniaturized handheld confocal microscopy for neurosurgery: results in an experimental glioblastoma model. Neurosurgery. 2010 Feb;66(2):410-7; discussion 417-8. doi: 10.1227/01.NEU.0000365772.66324.6F. PubMed PMID: 20087141.

6: Giller CA, Liu H, German DC, Kashyap D, Dewey RB. A stereotactic near-infrared probe for localization during functional neurosurgical procedures: further experience. J Neurosurg. 2009 Feb;110(2):263-73. doi: 10.3171/2008.8.JNS08728. PubMed PMID: 19012484.

7: Nowacki P, Tabaka J, Jezewski D, Honczarenko K. [Diagnosis of brain gliomas in stereotactic biopsy assisted by optical neuro-navigation system]. Neurol Neurochir Pol. 2004 Jan-Feb;38(1):3-8. Polish. PubMed PMID: 15049161.

8: Duffner F, Dauber W, Skalej M, Grote EH. A new endoscopic tool for the CRW Stereotactic System. Stereotact Funct Neurosurg. 1996-1997;67(3-4):213-7. PubMed PMID: 9311078.

9: Haglund MM, Hochman DW, Spence AM, Berger MS. Enhanced optical imaging of rat gliomas and tumor margins. Neurosurgery. 1994 Nov;35(5):930-40; discussion 940-1. PubMed PMID: 7838344.

1: Pillai A, Ratnathankom A, Ramachandran SN, Udayakumaran S, Subhash P, Krishnadas A. Expanding the Spectrum of Robotic Assistance in Cranial Neurosurgery. Oper Neurosurg (Hagerstown). 2018 Sep 7. doi: 10.1093/ons/opy229. [Epub ahead of print] PubMed PMID: 30203040.

2: Markwardt NA, Stepp H, Franz G, Sroka R, Goetz M, Zelenkov P, Rühm A. Remission spectrometry for blood vessel detection during stereotactic biopsy of brain tumors. J Biophotonics. 2017 Aug;10(8):1080-1094. doi: 10.1002/jbio.201600193. Epub 2016 Oct 7. PubMed PMID: 27714967.

3: Broadbent B, Tseng J, Kast R, Noh T, Brusatori M, Kalkanis SN, Auner GW. Shining light on neurosurgery diagnostics using Raman spectroscopy. J Neurooncol. 2016 Oct;130(1):1-9. Epub 2016 Aug 13. Review. PubMed PMID: 27522510.

4: Markwardt NA, Haj-Hosseini N, Hollnburger B, Stepp H, Zelenkov P, Rühm A. 405 nm versus 633 nm for protoporphyrin IX excitation in fluorescence-guided stereotactic biopsy of brain tumors. J Biophotonics. 2016 Sep;9(9):901-12. doi: 10.1002/jbio.201500195. Epub 2015 Nov 13. PubMed PMID: 26564058.

5: Stevens OA, Hutchings J, Gray W, Vincent RL, Day JC. Miniature standoff Raman probe for neurosurgical applications. J Biomed Opt. 2016 Aug 1;21(8):87002. doi: 10.1117/1.JBO.21.8.087002. PubMed PMID: 27533445.

6: Ragel BT, Ryken TC, Kalkanis SN, Ziu M, Cahill D, Olson JJ. The role of biopsy in the management of patients with presumed diffuse low grade glioma: A systematic review and evidence-based clinical practice guideline. J Neurooncol. 2015 Dec;125(3):481-501. doi: 10.1007/s11060-015-1866-2. Epub 2015 Nov 3. Review.

PubMed PMID: 26530259.

7: Oppido PA, Fiorindi A, Benvenuti L, Cattani F, Cipri S, Gangemi M, Godano U, Longatti P, Mascari C, Morace E, Tosatto L. Neuroendoscopic biopsy of ventricular tumors: a multicentric experience. Neurosurg Focus. 2011 Apr;30(4):E2. doi: 10.3171/2011.1.FOCUS10326. PubMed PMID: 21456929.

8: Frati A, Pichierri A, Bastianello S, Raco A, Santoro A, Esposito V, Giangaspero F, Salvati M. Frameless stereotactic cerebral biopsy: our experience in 296 cases. Stereotact Funct Neurosurg. 2011;89(4):234-45. doi: 10.1159/000325704. Epub 2011 Jul 21. PubMed PMID: 21778794.

9: Tian ZM, Wang YM, Yu X, Zhao QJ, Hui R, Liu R, Li ZC. [Clinical experience of stereotactic biopsy for the brain lesions]. Zhonghua Wai Ke Za Zhi. 2010 Oct 1;48(19):1459-62. Chinese. PubMed PMID: 21176652.

10: Chernov MF, Muragaki Y, Ochiai T, Taira T, Ono Y, Usukura M, Maruyama T, Nakaya K, Nakamura R, Iseki H, Kubo O, Hori T, Takakura K. Spectroscopy-supported frame-based image-guided stereotactic biopsy of parenchymal brain lesions: comparative evaluation of diagnostic yield and diagnostic accuracy. Clin Neurol Neurosurg. 2009 Jul;111(6):527-35. doi: 10.1016/j.clineuro.2009.03.006. Epub 2009 May 7. PubMed PMID: 19427112.

11: Giller CA, Liu H, German DC, Kashyap D, Dewey RB. A stereotactic near-infrared probe for localization during functional neurosurgical procedures: further experience. J Neurosurg. 2009 Feb;110(2):263-73. doi: 10.3171/2008.8.JNS08728. PubMed PMID: 19012484.

12: Herminghaus S, Dierks T, Pilatus U, Möller-Hartmann W, Wittsack J, Marquardt G, Labisch C, Lanfermann H, Schlote W, Zanella FE. Determination of histopathological tumor grade in neuroepithelial brain tumors by using spectral pattern analysis of in vivo spectroscopic data. J Neurosurg. 2003 Jan;98(1):74-81. PubMed PMID: 12546355.

13: Samadani U, Judy KD. Stereotactic brainstem biopsy is indicated for the diagnosis of a vast array of brainstem pathology. Stereotact Funct Neurosurg. 2003;81(1-4):5-9. PubMed PMID: 14742957.

14: Koljenović S, Choo-Smith LP, Bakker Schut TC, Kros JM, van den Berge HJ, Puppels GJ. Discriminating vital tumor from necrotic tissue in human glioblastoma tissue samples by Raman spectroscopy. Lab Invest. 2002 Oct;82(10):1265-77. PubMed PMID: 12379761.

1: Broadbent B, Tseng J, Kast R, Noh T, Brusatori M, Kalkanis SN, Auner GW. Shining light on neurosurgery diagnostics using Raman spectroscopy. J Neurooncol.

2016 Oct;130(1):1-9. Epub 2016 Aug 13. Review. PubMed PMID: 27522510.

2: Stevens OA, Hutchings J, Gray W, Vincent RL, Day JC. Miniature standoff Raman probe for neurosurgical applications. J Biomed Opt. 2016 Aug 1;21(8):87002. doi: 10.1117/1.JBO.21.8.087002. PubMed PMID: 27533445.

1: Kiesel B, Millesi M, Woehrer A, Furtner J, Bavand A, Roetzer T, Mischkulnig M, Wolfsberger S, Preusser M, Knosp E, Widhalm G. 5-ALA-induced fluorescence as a marker for diagnostic tissue in stereotactic biopsies of intracranial lymphomas: experience in 41 patients. Neurosurg Focus. 2018 Jun;44(6):E7. doi: 10.3171/2018.3.FOCUS1859. PubMed PMID: 29852770.

2: Bowden SG, Neira JA, Gill BJA, Ung TH, Englander ZK, Zanazzi G, Chang PD, Samanamud J, Grinband J, Sheth SA, McKhann GM 2nd, Sisti MB, Canoll P, D'Amico RS, Bruce JN. Sodium Fluorescein Facilitates Guided Sampling of Diagnostic Tumor Tissue in Nonenhancing Gliomas. Neurosurgery. 2018 May 1;82(5):719-727. doi: 10.1093/neuros/nyx271. PubMed PMID: 28645214.

3: Haj-Hosseini N, Richter JCO, Milos P, Hallbeck M, Wårdell K. 5-ALA fluorescence and laser Doppler flowmetry for guidance in a stereotactic brain tumor biopsy. Biomed Opt Express. 2018 Apr 20;9(5):2284-2296. doi: 10.1364/BOE.9.002284. eCollection 2018 May 1. PubMed PMID: 29760987; PubMed

Central PMCID: PMC5946788.

4: Thien A, Han JX, Kumar K, Ng YP, Rao JP, Ng WH, King NKK. Investigation of the usefulness of fluorescein sodium fluorescence in stereotactic brain biopsy. Acta Neurochir (Wien). 2018 Feb;160(2):317-324. doi: 10.1007/s00701-017-3429-0. Epub 2017 Dec 23. PubMed PMID: 29275519.

5: Catapano G, Sgulò FG, Seneca V, Iorio G, de Notaris M, di Nuzzo G. Fluorescein-assisted stereotactic needle biopsy of brain tumors: a single-center experience and systematic review. Neurosurg Rev. 2018 Jan 30. doi: 10.1007/s10143-018-0947-z. [Epub ahead of print] Review. PubMed PMID: 29383601.

6: Thien A, Rao JP, Ng WH, King NK. The Fluoropen: a simple low-cost device to detect intraoperative fluorescein fluorescence in stereotactic needle biopsy of brain tumors. Acta Neurochir (Wien). 2017 Feb;159(2):371-375. doi: 10.1007/s00701-016-3041-8. Epub 2016 Dec 10. PubMed PMID: 27943078.

7: Markwardt NA, Haj-Hosseini N, Hollnburger B, Stepp H, Zelenkov P, Rühm A. 405 nm versus 633 nm for protoporphyrin IX excitation in fluorescence-guided stereotactic biopsy of brain tumors. J Biophotonics. 2016 Sep;9(9):901-12. doi: 10.1002/jbio.201500195. Epub 2015 Nov 13. PubMed PMID: 26564058.

8: Cordone I, Masi S, Carosi M, Vidiri A, Marchesi F, Marino M, Telera S, Pasquale A, Mengarelli A, Conti L, Pescarmona E, Pace A, Carapella CM. Brain stereotactic biopsy flow cytometry for central nervous system lymphoma characterization: advantages and pitfalls. J Exp Clin Cancer Res. 2016 Aug 27;35(1):128. doi: 10.1186/s13046-016-0404-1. PubMed PMID: 27567676; PubMed Central PMCID: PMC5002320.

9: Rühm A, Göbel W, Sroka R, Stepp H. ICG-assisted blood vessel detection during stereotactic neurosurgery: simulation study on excitation power limitations due to thermal effects in human brain tissue. Photodiagnosis Photodyn Ther. 2014 Sep;11(3):307-18. doi: 10.1016/j.pdpdt.2014.03.007. Epub 2014 Mar 31. PubMed PMID: 24699453.

10: Rey-Dios R, Hattab EM, Cohen-Gadol AA. Use of intraoperative fluorescein sodium fluorescence to improve the accuracy of tissue diagnosis during stereotactic needle biopsy of high-grade gliomas. Acta Neurochir (Wien). 2014 Jun;156(6):1071-5; discussion 1075. doi: 10.1007/s00701-014-2097-6. Epub 2014 Apr 27. PubMed PMID: 24770732.

11: Piquer J, Llácer JL, Rovira V, Riesgo P, Rodriguez R, Cremades A. Fluorescence-guided surgery and biopsy in gliomas with an exoscope system. Biomed Res Int. 2014;2014:207974. doi: 10.1155/2014/207974. Epub 2014 May 21. PubMed PMID: 24971317; PubMed Central PMCID: PMC4055357.

12: Göbel W, Brucker D, Kienast Y, Johansson A, Kniebühler G, Rühm A, Eigenbrod S, Fischer S, Goetz M, Kreth FW, Ehrhardt A, Stepp H, Irion KM, Herms J. Optical needle endoscope for safe and precise stereotactically guided biopsy sampling in neurosurgery. Opt Express. 2012 Nov 19;20(24):26117-26. doi: 10.1364/OE.20.026117. PubMed PMID: 23187467.

13: Widhalm G, Minchev G, Woehrer A, Preusser M, Kiesel B, Furtner J, Mert A, Di Ieva A, Tomanek B, Prayer D, Marosi C, Hainfellner JA, Knosp E, Wolfsberger S. Strong 5-aminolevulinic acid-induced fluorescence is a novel intraoperative marker for representative tissue samples in stereotactic brain tumor biopsies. Neurosurg Rev. 2012 Jul;35(3):381-91; discussion 391. doi: 10.1007/s10143-012-0374-5. Epub 2012 Mar 10. PubMed PMID: 22407140.

14: von Campe G, Moschopulos M, Hefti M. 5-Aminolevulinic acid-induced protoporphyrin IX fluorescence as immediate intraoperative indicator to improve the safety of malignant or high-grade brain tumor diagnosis in frameless stereotactic biopsies. Acta Neurochir (Wien). 2012 Apr;154(4):585-8; discussion 588. doi: 10.1007/s00701-012-1290-8. PubMed PMID: 22297399; PubMed Central PMCID: PMC3308005.

15: Arita H, Kinoshita M, Kagawa N, Fujimoto Y, Kishima H, Hashimoto N, Yoshimine T. ^{11}C-methionine uptake and intraoperative 5-aminolevulinic acid-induced fluorescence as separate index markers of cell density in glioma: a stereotactic

image-histological analysis. Cancer. 2012 Mar 15;118(6):1619-27. doi: 10.1002/cncr.26445. Epub 2011 Aug 11. PubMed PMID: 21837671.

16: Yamaguchi F, Takahashi H, Teramoto A. Photodiagnosis for frameless stereotactic biopsy of brain tumor. Photodiagnosis Photodyn Ther. 2007 Mar;4(1):71-5. doi: 10.1016/j.pdpdt.2006.09.005. Epub 2006 Nov 3. PubMed PMID: 25047195.
